# Analysis of environmental factors using AI and ML methods

**DOI:** 10.1038/s41598-022-16665-7

**Published:** 2022-08-02

**Authors:** Mohd Anul Haq, Ahsan Ahmed, Ilyas Khan, Jayadev Gyani, Abdullah Mohamed, El-Awady Attia, Pandian Mangan, Dinagarapandi Pandi

**Affiliations:** 1 Department of Computer Science, College of Computer and Information Sciences, Majmaah University, Al Majmaah, 11952 Saudi Arabia; 2 Department of Information Technology, College of Computer and Information Sciences, Majmaah University, Al Majmaah, 11952 Saudi Arabia; 3 Basic Engineering Sciences Department, College of Engineering, Majmaah University, Al Majmaah, 11952 Saudi Arabia; 4 University Research Centre, Future University in Egypt, New Cairo, 11745 Egypt; 5 Department of Industrial Engineering, College of Engineering, Prince Sattam Bin Abdulaziz University, Al Kharj, 16273 Saudi Arabia; 6 Mechanical Engineering Department, Faculty of Engineering (Shoubra), Benha University, Cairo, Egypt; 7 Amnex Infotechnologies Pvt. Ltd, Ahmadabad, 380052 Gujarat India; 8 School of Civil Engineering, Vellore Institute of Technology, Chennai, 600127 India

**Keywords:** Climate change, Environmental impact, Climate sciences, Environmental sciences

## Abstract

The main goal of this research paper is to apply a deep neural network model for time series forecasting of environmental variables. Accurate forecasting of snow cover and NDVI are important issues for the reliable and efficient hydrological models and prediction of the spread of forest. Long Short Term Memory (LSTM) model for the time series forecasting of snow cover, temperature, and normalized difference vegetation index (NDVI) are studied in this research work. Artificial neural networks (ANN) are widely used for forecasting time series due to their adaptive computing nature. LSTM and Recurrent neural networks (RNN) are some of the several architectures provided in a class of ANN. LSTM is a kind of RNN that has the capability of learning long-term dependencies. We followed a coarse-to-fine strategy, providing reviews of various related research materials and supporting it with the LSTM analysis on the dataset of Himachal Pradesh, as gathered. Environmental factors of the Himachal Pradesh region are forecasted using the dataset, consisting of temperature, snow cover, and vegetation index as parameters from the year 2001–2017. Currently, available tools and techniques make the presented system more efficient to quickly assess, adjust, and improve the environment-related factors analysis.

## Introduction

Extracting patterns from time-series data is significant in forecasting future development^1^. Many researchers are working on this topic for several years to improve the accuracy and efficiency of time series modeling and forecasting. This research work proposed deep learning techniques for environmental factors future forecasting. A comparison of the performance of time series forecasting using ANN and LSTM is done in this research work. In the past, time series have been forecasted by using RNN, e.g., for market prediction, weather forecasting^2^, and forecasting of network traffic^4^. The sequences of previous time steps are used to predict the next or future step using RNN. The three gates used in RNN are suited for classifying, processing, and making forecasting a given time series data, since there can be gaps in between important events which are unknown in time series. Moreover, each RNN can also be trained with various sets of hyperparameters i.e., the number of layers, the number of neurons, or can be the number of past time steps.

The main aim of this paper is to present LSTM forecasting, that supports the performance comparison of individual networks on training data and test data in the context of environmental factors. The major novelty of the present research is to develop and implement LSTM models on several combinations of parameters and rigorous hyperparameter tuning to increase the efficiency and stability of the proposed model. Another novelty of the present study was the processing and extraction of the environmental variables dataset based on bulk processing alongside the future forecasting of environmental variables using the moving-forward window technique for future seven years. The present investigation contributes to the environmental modeling knowledge based on three important environmental variables and their associated responses. The present study highlights the effect of changing climate on snow cover and vegetation^3^. Additionally, the current investigation performed statistical trends change analysis to know the temporal influence of these three important environmental parameters which serve as important indicators of climate change effects.

### Related works

Many important initiatives to solve the challenge of weather forecasting have been published in the last decade. For time-series datasets such as signals, text, etc., an LSTM is a higher-ranking method for investigating temporal patterns in neural networks^5^. An LSTM defeats the vanishing gradient problem in RNN to gather information on long-term dependencies in the time series dataset using the memory cells and gates^6^. Some experimental outcomes showed that the LSTM was more precise in terms of values than other machine learning models.

ANN are highly parallel systems with the interconnection of large numbers of simple processors. ANN works fine for applications that are based on Optical Character Recognition (OCR). But no convincing evidence for their superiority over conventional statistical pattern classifiers^7^ has been found. In ANN the input signal is a real number at the start of the association between the artificial neurons and the output is calculated by some non-linear functions of the sum of its inputs. The connections between artificial neurons are known as edges and these have weights that get readjusted as the learning procedure proceeds. The strength of the signal is dependent on the weight of the edges. The signal travel from one layer to other if it crosses the threshold that ANN has. Usually, ANN is aggregated into numerous layers for performing various transformations on input signals. The output layer generates results after traversing the input signal multiple times through the numerous layers of ANN^8^.

A neural network was trained using the gas production generated dataset to forecast the production of gas. To achieve optimum gas production performance, the regressor output was subjected to three algorithms: cohort intelligence (CI), multi-cohort intelligence (multi-CI), and teaching learning-based optimization (TLBO) algorithms. The findings showed that in comparison to CI, genetic algorithm, and particle swarm optimization; TLBO algorithms and multi-CI, converge at the global best position more frequently with a 95% success rate^9^. A proxy modeling technique with mechanical restrictions and intelligent modeling strategies were used to present a smart unconventional enhanced oil recovery (EOR) performance evaluation tool. Deep Neural Network models with various key parameters like matrix and hydraulic fracture permeability contrast, and average reservoir pressure was trained. To predict recovery performance over two different time frames, a total of four DNN models were created. For EOR application forecasts, the reservoir and hydraulic fracture characteristics must be equivalent. Operators will be able to optimize their project planning and screening with this approach^10^. AI and ML techniques were used for analyzing production performance. Exploration and production of hydrocarbons is an unceasing operation that generates a large amount of information from both subsurface and surface facilities. The availability of such a large dataset that continues to grow over time improves computing capabilities and performance accuracy through data-driven applications using AI and ML concepts. ANN, as well as supervised and unsupervised approaches, can be used in the ML approach. Support vector machine (SVM), Random Forest (RF), clustering methods, and artificial network-based architecture are some of the other machine learning approaches which can be used for finding more efficient predictive outputs^11^. With the increase of demand in shale reservoirs, a review study was done by Syed^13^ to model petrophysical and geo-mechanical properties using ML techniques. In continuation of this, another study on recent ML based models for the estimation of production performance of shale gas reservoirs was done by Syed^12^. Various key parameters and ML algorithms used to forecast the shale gas reservoir’s production behavior were discussed along with their advantages and disadvantages.

In the previous related work, the LSTM and ANN models are used for time series forecasting of air pollution^14^, weather prediction^6^, and rainfall-runoff simulation^15^, and resulted in higher efficiency of the model. Making use of LSTM, a green-house climate prediction model (GCP_lstm) was proposed for predicting the effect of six climate factors (i.e., temperature, light, humidity, the concentration of CO_2,_ soil temperature, and soil humidity) on the growth of crops. A time sliding window was added to record climate changes for every minute. The model was applied to the datasets of three vegetables and attained good accuracy in comparison to other models. The model was robust and achieve the best performance at a fixed time window of 5 min. On increasing the window size, the performance of the model decreases^16^. An LSTM-based model was used by Liu^17,18^ for predicting the POI category. Weather forecasting data was used to predict hourly day-ahead irradiance using a unique solar prediction technique along with LSTM. The model considers the dependence of alternating hours of a day. The suggested approach shows less overfitting and better generalization capabilities than the backpropagation algorithm (BPNN). The suggested LSTM algorithm reduces the prediction RMSE by 42.9 percent when compared to BPNN^19^.

An LSTM and Transudative LSTM (T-LSTM) were used to prepare a model for a weather application to forecast time series data. For the regression problem, a quadratic cost function was used. The cosine similarity between the training and test samples was used to explore two weighting strategies. The experiments are carried out across two separate periods of a year to assess the efficacy of the suggested approach under various weather conditions. T-LSTM performs better in the prediction task, according to the findings^20^. Making use of a single layer and multilayer LSTM model, a statistical model was developed for forecasting weather variables in the area of Indonesian airport, as well as to investigate the impact of intermediate weather variables on accuracy prediction. By incorporating intermediate variable signals into the memory block of LSTM, the suggested model was an expansion of the basic LSTM model. Visibility was employed as a predictor, and pressure, humidity, temperature, and dew point were used as intermediates. In this research work, the optimum LSTM model was multi-layer LSTM, and the pressure variable was the finest intermediate data^22^. A stacked LSTM model was implemented in a weather forecasting application. The first LSTM layer consists of distinct LSTM models per location and the second LSTM layer uses the hidden states of the first LSTM layer as an input. The experimental work shows that the prediction performance of the model based on stacked LSTM was improved using spatial information^26^.

### Study area map

**Figure 1:**
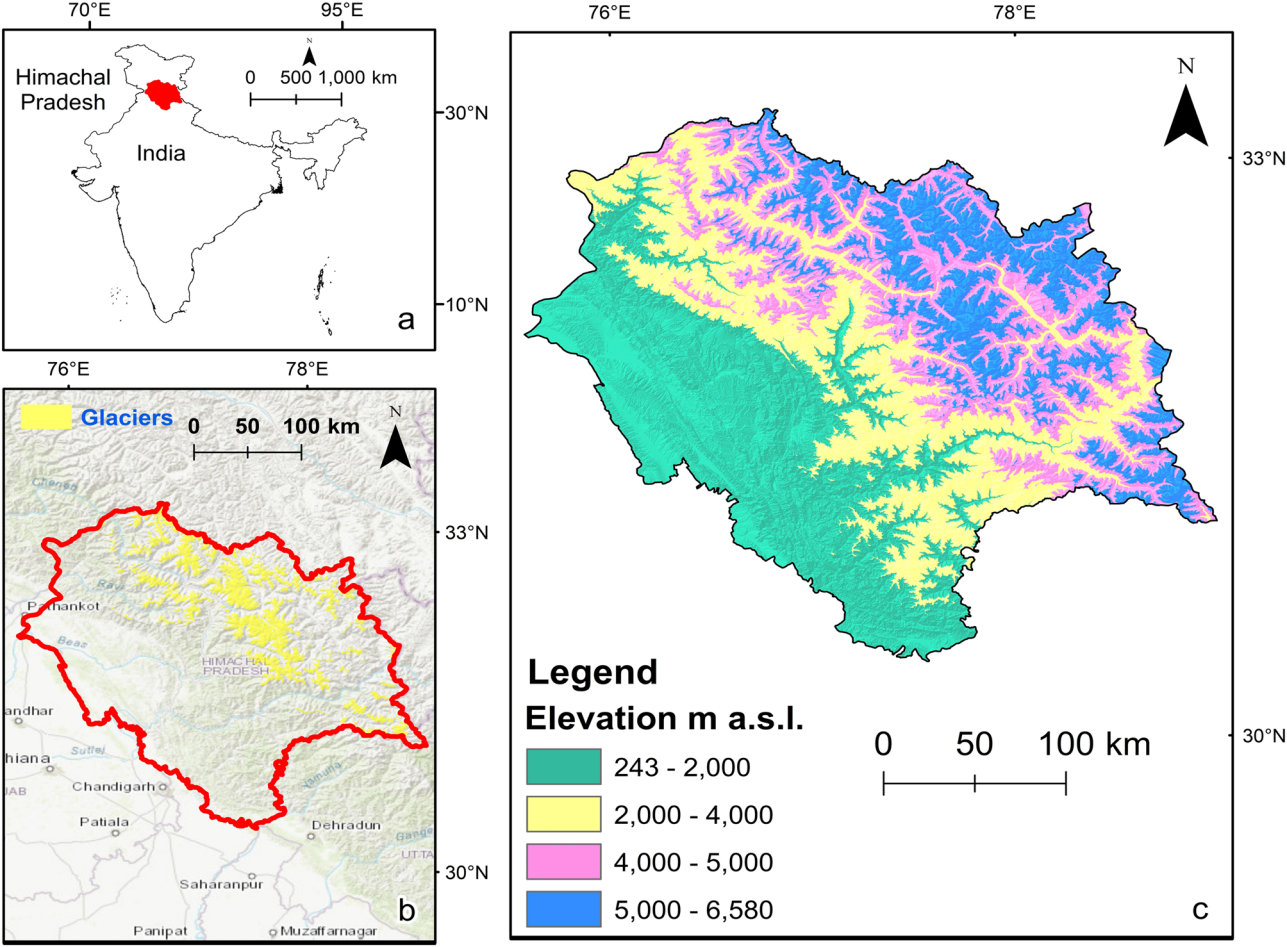
(**a**) Map of India. The region of Himachal Pradesh is highlighted in red. (**b**) The outline of the Himachal Pradesh region is highlighted in red. Glaciers based on Randolph Glacier Inventory Version6.0 (RGI 2017) within the state are highlighted in yellow. Background image: Topographic Map (ESRI2016- https://www.esri.com/) generated using ArcMap version 10.3. (**c**) Distribution of various elevation ranges within the region of Himachal Pradesh.

The data used for the model has been taken from the region mentioned in Fig. 1. Inventory (2017) (ESRI, Redlands, California). Figure 1a. represents the map of India, and the highlighted part shows the Himachal Pradesh region. Figure 1b. reflects the outline of the Himachal Pradesh region that was highlighted in red color and the yellow portion shows the glaciers within the state that are based on Randolph Glacier Inventory Version 6.0 (RGI 2017)^23^ and we consider glaciers as it represents high elevation and also, they are strong factors for climate distribution, as the study of this paper is about climate so the glaciers are included in the map. Figure 1c. shows the distribution of various elevation ranges within the region of Himachal Pradesh.

### Data used

Machine learning and deep learning techniques are more about training and testing the data to algorithms to perform various intensive tasks. However, there are many challenges in taking the right data to algorithms or cleaning unnecessary and error-prone data. With the use of uncleaned data, the models will take a much harder time capturing the actual important features in the exploration. In order, to fit the model well the data should be filtered and modified such that it is easier to explore, understand and extract features. The data used in this paper is from the Himachal Pradesh region and taken temperature in kelvin, snow cover, and vegetation in pixels from 2001 to 2017. These environmental factors play a significant role in hydrology and climate at both regional and global scales. Static threshold techniques had been used by many researchers to detect these factors, but it leads to an error such as improper classification of clouds and snow. Therefore, the data has been captured using a Moderate Resolution Spectroradiometer (MODIS) and collected week wise and later the data is averaged to a monthly format and used for this model.

## Methods

### Artificial neural networks (ANN)

It is a mathematical model that reproduces the operation of a biological neuron. ANN is important for discovering the hidden patterns in complex and nonlinear structured data^1^. ANN learns the function of f(x) = y, where x is an input and y is an output variable. Initially, ANN architecture is a Multi-layer Perceptron (MLP)^24^, a neural network having a fully connected architecture that shows the quality output that has been widely used by the researchers for pattern detection. But, for highly complex data, the MLP architecture may be inefficient to identify all the conditions^14^. Traditional feedforward networks are composed of three parts: the input layer is the first layer that takes input signal x, the second layer is the hidden layer(s) and the third layer is an output layer. Each layer consists of several neurons along with their weights. The weights of neurons are used in the training period to reduce the error between the function f(x) and the target output y^1^. Various new ANN architectures have been proposed and developed by researchers and scientists working in the field of ANN.

### Deep learning method

At present, deep learning methods are used to solve the problem and give better performance in comparison to past machine learning methods. Mainly they are used in the fields of natural language processing tasks^25^, speech recognition, and image capturing. Solving the problem by deep learning methods requires designing an architecture similar to a neural network, but designing an architecture requires many hidden layers and a huge data set then it extracts more features used for forecasting and it reduces the need for feature engineering^27^. The data of environmental factors are generally in sequential format and to process this time series data, the frequency of using LSTM and Recurrent Neural Network (RNN) is higher in comparison to other neural networks.

### Recurrent neural networks (RNN)

RNN is a type of neural network that does not propagate the input signal straight through the network. It takes the previous step’s output as an input for the current step. RNN networks contain infinite loops for information persistence^6^. RNN has memory and is recommended for solving problems with a temporal dimension.

For a given sequence of inputs (× 1, × 2, × 3, …, xn), RNN computes (y1,y2,…,yn) as a sequence of outputs by iterating over the Eq. (1)^6^.

1 $${h}_{t}=sigm\left({w}^{hx}{X}_{t}+{w}^{hh}{h}_{t-1}\right){y}_{t}={w}^{yh}{h}_{t}$$

The RNN can map sequence to sequence whenever the alignment between the input and the output is known upfront.

### The problem of long-term dependencies

Developing a weather forecasting model using the past years’ datasets is not a good choice. The latest weather data is more crucial for accurate forecasting and, one might have to look at the latest weather-relevant information for performing the forecasting^6^. However, in some cases, past data can also support the model to capture movements and patterns that new data fails to exhibit. Unfortunately, due to the gap between the recent and the past information the traditional RNNs stop learning to connect the information, and give rise to the problem of Long-Term Dependencies. LSTM is a type of RNN that includes a memory cell in LSTM blocks to retain the information for a long duration of time^28^. Additionally, a set of gates are also used for memory manipulations. This architecture helps to learn longer-term dependencies.

### Long short-term memory networks (LSTM)

The LSTM architecture is a type of recurrent neural network (RNN) that was created to model temporal sequences. Long-range dependencies in LSTM make it more accurate than traditional RNNs. LSTMs are explicitly designed to evade the problem of vanishing gradient problems from traditional RNN by enabling them to learn long-term dependencies^28^. LSTM does not require any adjustments of weight and hence the complexity of updating the weights is minimal. LSTM is one of the popular RNNs that is used for various tasks including time series forecasting, speech recognition, controlling robot, text generation, etc. LSTM architecture includes a memory cell as a part of the LSTM block and three regulators known as gates, used for the propagation of information inside or outside the LSTM unit: an input gate, output gate, and forget gate^5^. Multiple variations of LSTM units are available, some units do not include one or more of these gates or use other gates. The new value flows into the cell are controlled by the input gate. The decision to keep the value in the cell is managed by the forget gate the output gate will decide which value within the cell is to be used for computing the output activation function of the LSTM unit which is a logistic function of LSTM^26^. In addition to in and out connections of the LSTM gates, there are some recurrent connections too. The operations of the logic gates are dependent on the weights of these connections that are learned during the training. The below mentioned are the Eqs. (2–7) of LSTM^10^.

Input node

2 $${g}^{\left(t\right)}=\mathrm{tanh}\left({W}_{gx}{\mathrm{x}}^{\left(t\right)}\boldsymbol{ }+\boldsymbol{ }\boldsymbol{ }{W}_{gh}{h}^{\left(t-1\right)}\boldsymbol{ }+\boldsymbol{ }{b}_{g}\right)$$

Input gate

3 $${i}^{\left(t\right)} =\upsigma \left({W}_{ix}{\mathrm{x}}^{\left(t\right)}+\boldsymbol{ }{W}_{ih}{h}^{\left(t-1\right)}\boldsymbol{ }+\boldsymbol{ }{b}_{i}\right)$$

Output gate

4 $${o}^{\left(t\right)} =\upsigma \left({W}_{ox}{\mathrm{x}}^{\left(t\right)}+\boldsymbol{ }{W}_{oh}{h}^{\left(t-1\right)}\boldsymbol{ }+\boldsymbol{ }{b}_{o}\right)$$

Cell state

5 $$\left( t \right) = g^{\left( t \right)} { } \odot \user2{ }i^{\left( t \right)} + (t - 1) \odot o^{\left( t \right)}$$

Hidden gate

6 $$h^{\left( t \right)} = \tanh \left( {{\text{s}}^{\left( t \right)} } \right)\user2{ } \odot o^{\left( t \right)}$$

Output layer

7 $${y}^{\left(t\right)} = \left({W}_{hy}{h}^{\left(t\right)}+\boldsymbol{ }{b}_{y}\right)$$

## Methodology

**Figure 2:**
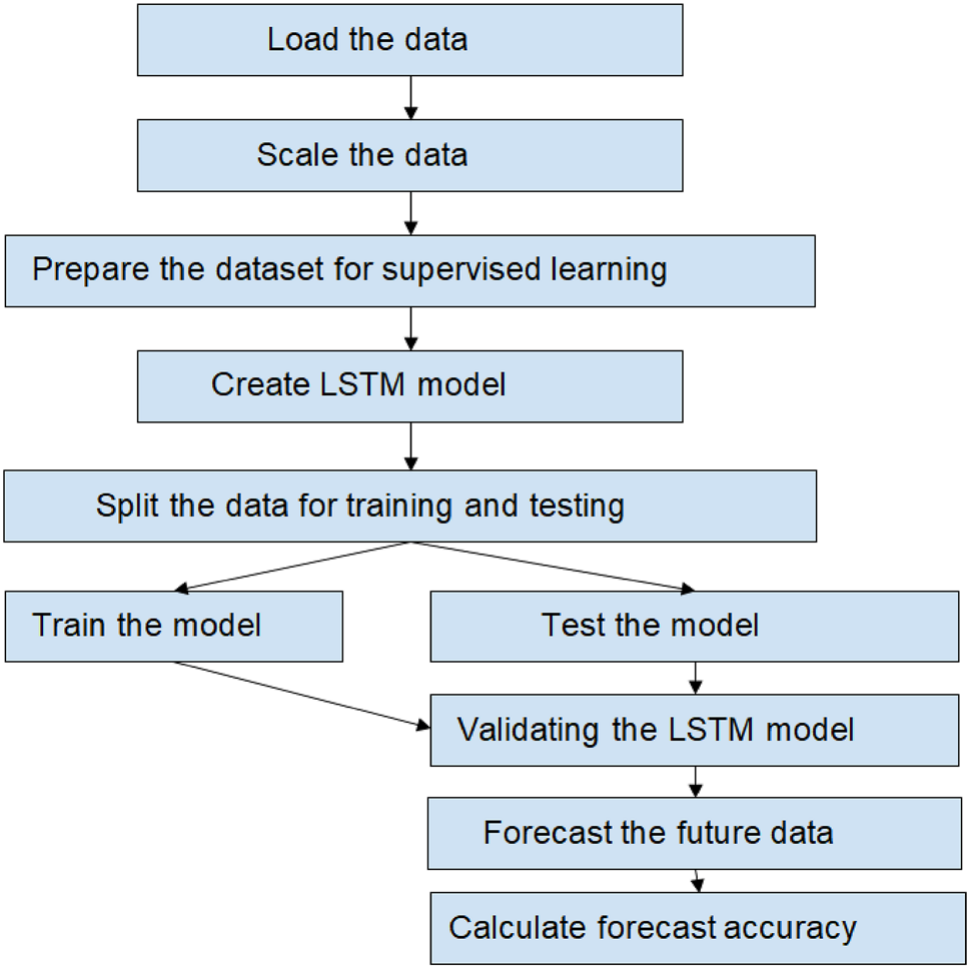
Flowchart of implementing LSTM model.

An LSTM network is a type of RNN having cells blocks and these cells are manipulated using the components called input gate, forget gate, and output gate. The functionality of forget gate enables it to forget the irrelevant data, the input is given to the sigmoid layer that decides which part from old information must be removed, and in the next step, the input gate takes the decision to store information from the new input in the cell block and later the output gate will decide the output of the network. In this paper, the model initializes with loading the dataset and the data has been scaled to 0–1 using a min–max scaler because the dataset contains features that vary highly in magnitudes, range, and units. Later, the data has been prepared in a way that suits well for supervised learning. And in the next step optimization of hyperparameters is performed such as neurons, optimizers, epochs, etc. that decides the accuracy of the model if the hyperparameters are not tuned appropriately then the model may result in underfitting or overfitting. Dropouts were implemented to avoid the issue of overfitting in the present investigation. After tuning the parameters, the dataset is divided for training and testing the model. Later, the model applies the moving-forward window technique to forecast the future values and the forecasted and actual values are compared to estimate the R-square value of the model. Figure 2 display the flowchart of implementing the LSTM model.

## Results

**Figure 3:**
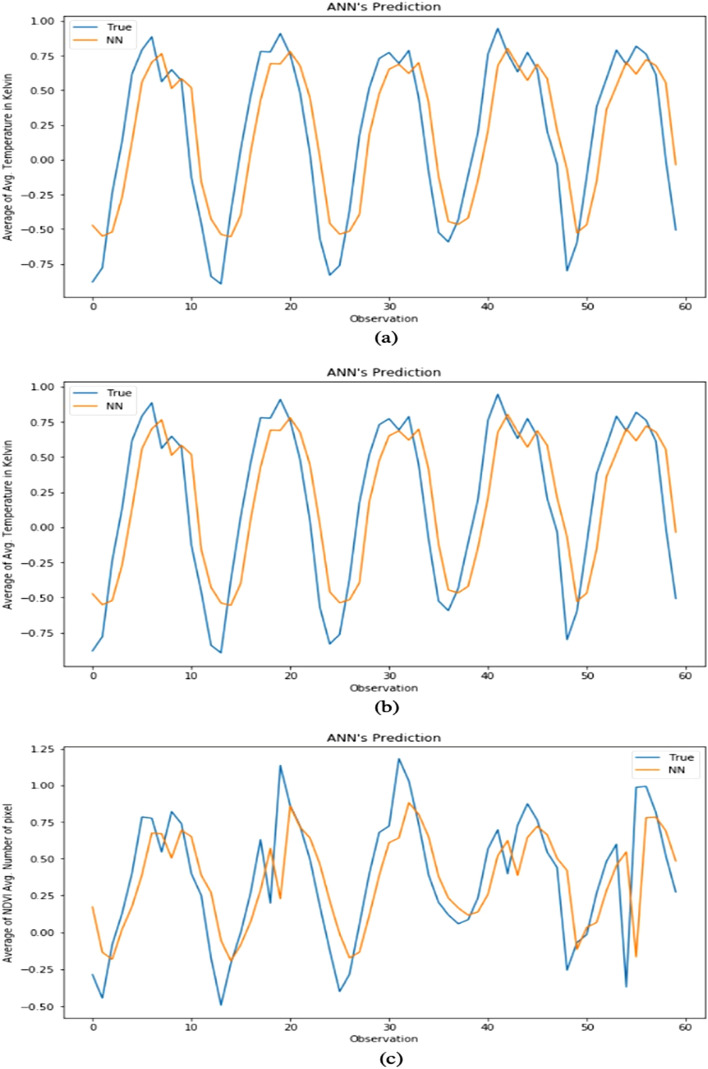
Forecasting using a single feature ANN model. (**a**) Temperature. (**b**) Snow cover. (**c**) NDVI.

**Figure 4:**
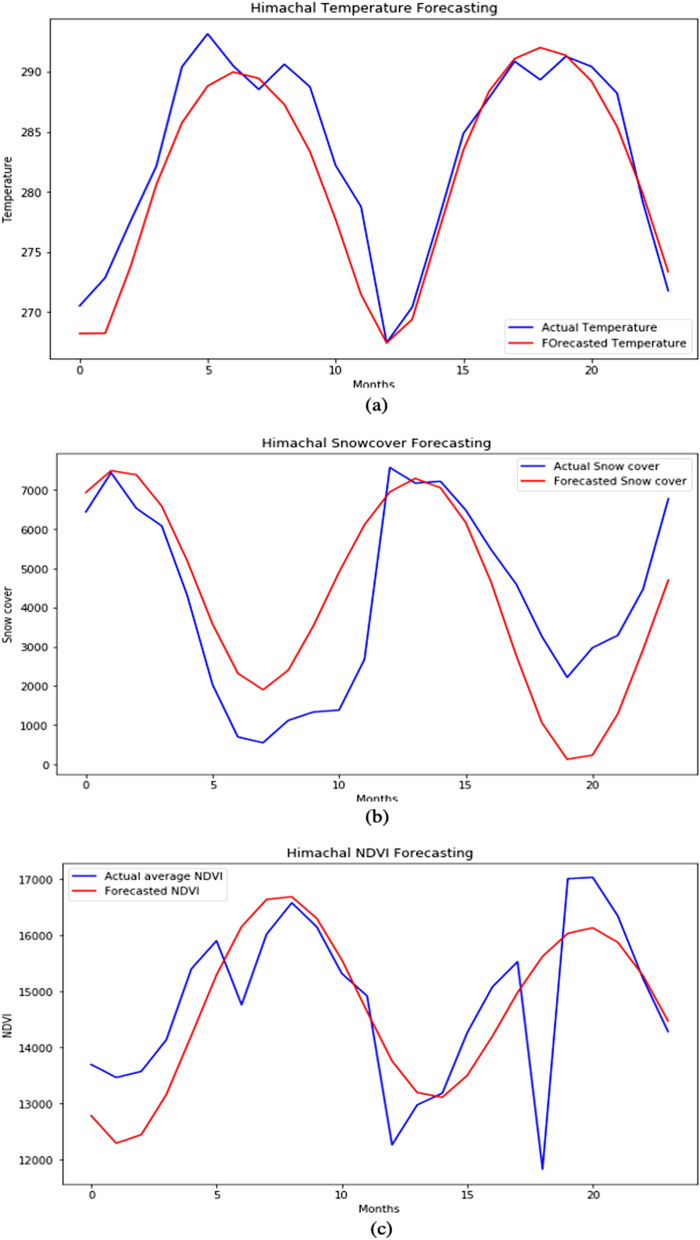
Forecasting using a single feature LSTM model. (**a**) Temperature. (**b**) Snow cover. (**c**) NDVI.

Initially, we performed validation using ANN and LSTM to choose the better model for our data. Both the ANN and LSTM model are trained and tested with data using one input and one output parameter. The R-square values obtained for temperature, snow cover and NDVI using ANN are 0.681, 0.66, 0.404 (Fig. 3a–c) and 0.89, 0.674, 0.48 using LSTM (Fig. 4a–c) respectively.

From the above results, we can conclude that deep learning provides a lot of advantages for time series forecasting, like automatic learning of temporal dependence and handling of temporal structures like features and seasonality. It can be applied for the multiple numbers of inputs used for a mapping function and thereby supporting multivariate time series forecasting. With its effective way of extracting patterns from the long sequences of input data, it can be perfectly applicable to deal with the massive amounts of data, complex parameters, and multi-step actions. To increase the accuracy of LSTM we tried two input and one output model with different hyperparameters.

### LSTM implementation

In this section, the LSTM was applied for different models to forecast time series data to find which model is best suited (which are having high r-square values) for individual environmental factors.

#### Using LSTM model with one input and one output feature

Here the LSTM model was implemented for forecasting one factor (example temperature) using the same factor as the input feature. Taking the data of each parameter individually from 2001 to 2015 for training the model and the next 2 years of data for testing the model. A dense layer is added at the end of the model to make it more robust. Since only a single value is to be forecasted in the output, only one neuron will be set in the dense layer. We have used Mean squared error as a loss function and Adam optimizer is used to optimize the algorithm. The figures mentioned below show the testing results of the temperature, snow cover, and NDVI respectively with one feature. The R-square values of these results are 0.89 for temperature Fig. 4a, 0.674 for snow cover Fig. 4b, and 0.48 for NDVI Fig. 4c. If the R-square value is above 0.75 then it is considered a good model. But as mentioned it is only greater for temperature and not for snow cover and NDVI, so this model with one feature is best suited for temperature but not ideal for the other two parameters.

#### Using the LSTM model with two input and one output feature

LSTM can seamlessly encounter difficulties with multiple input parameters. All we’d like may be a 3D input vector that has to be fed into the input-form of the LSTM. So, if finding a way to change the input parameters to be represented in a 3D vector form, we can use LSTM. The parameters used i.e., temperature, snow cover, NDVI that are interrelated to each other. In the above method, the model takes each parameter individually for input as well as output and forecasts but the R-square values obtained are not efficient that we cannot accept the model. To increase the accuracy of the model, we have implemented the LSTM model with multiple combinations of these parameters as input and one output feature and forecasted accordingly to monthly, seasonal, and annual.

#### Using the LSTM model with one input and one output feature

**Figure 5:**
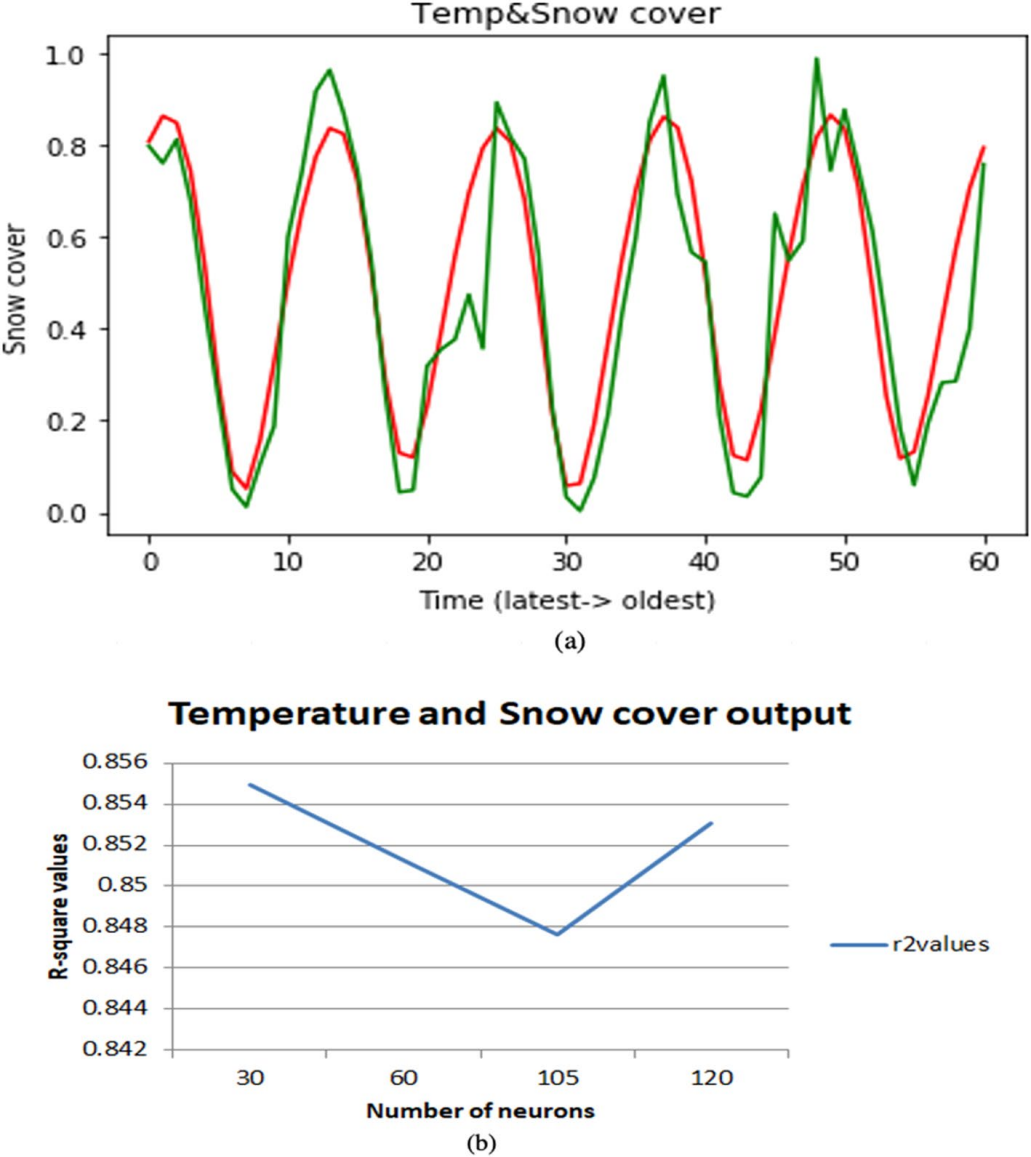
(**a**) Snow cover monthly as an output on multiple combinations. (**b**) Changing the number of neurons and best r-square at 30 neurons.

**Figure 6:**
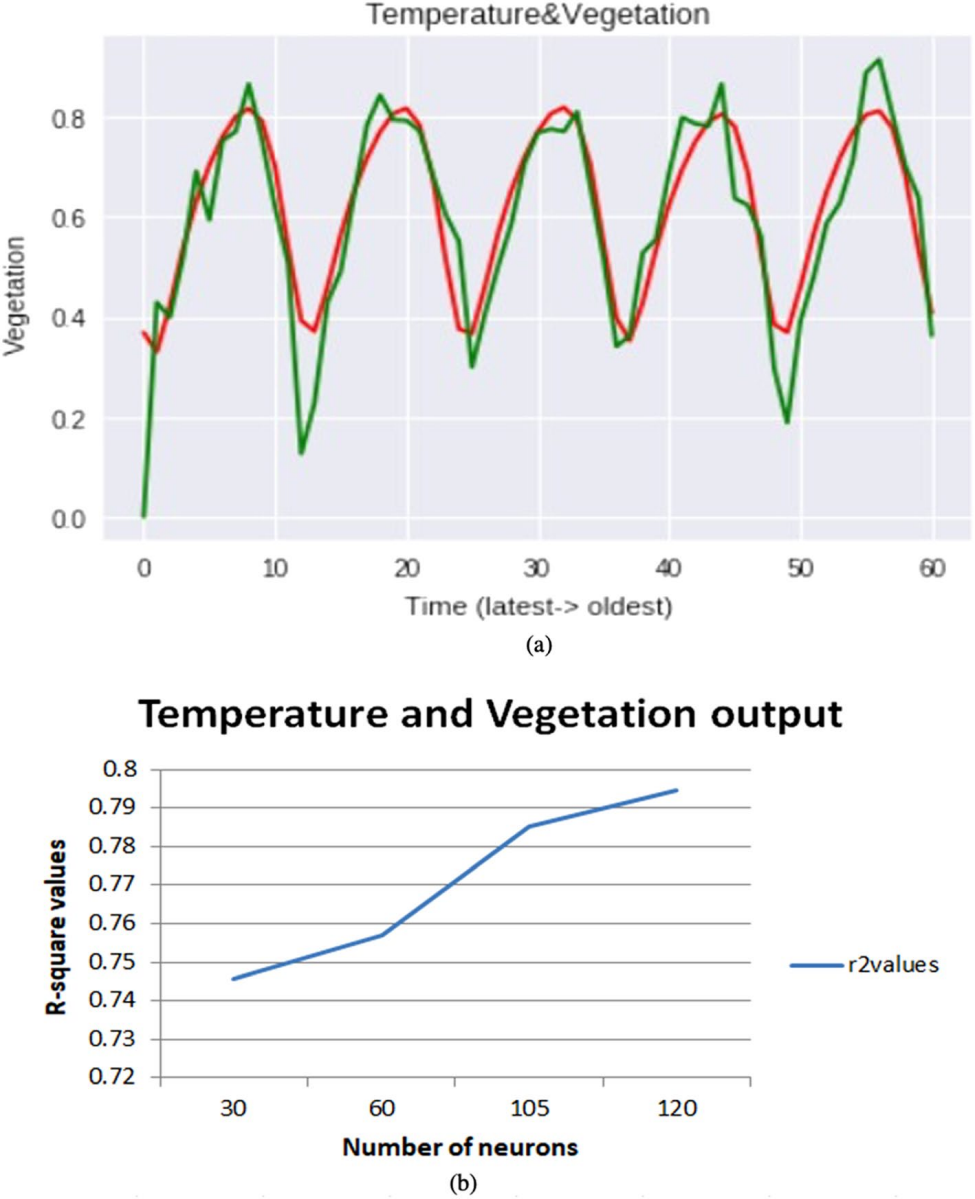
(**a**) Vegetation monthly as output on multiple combinations. (**b**) Changing the number of neurons and best r-square at 120 neurons.

**Figure 7:**
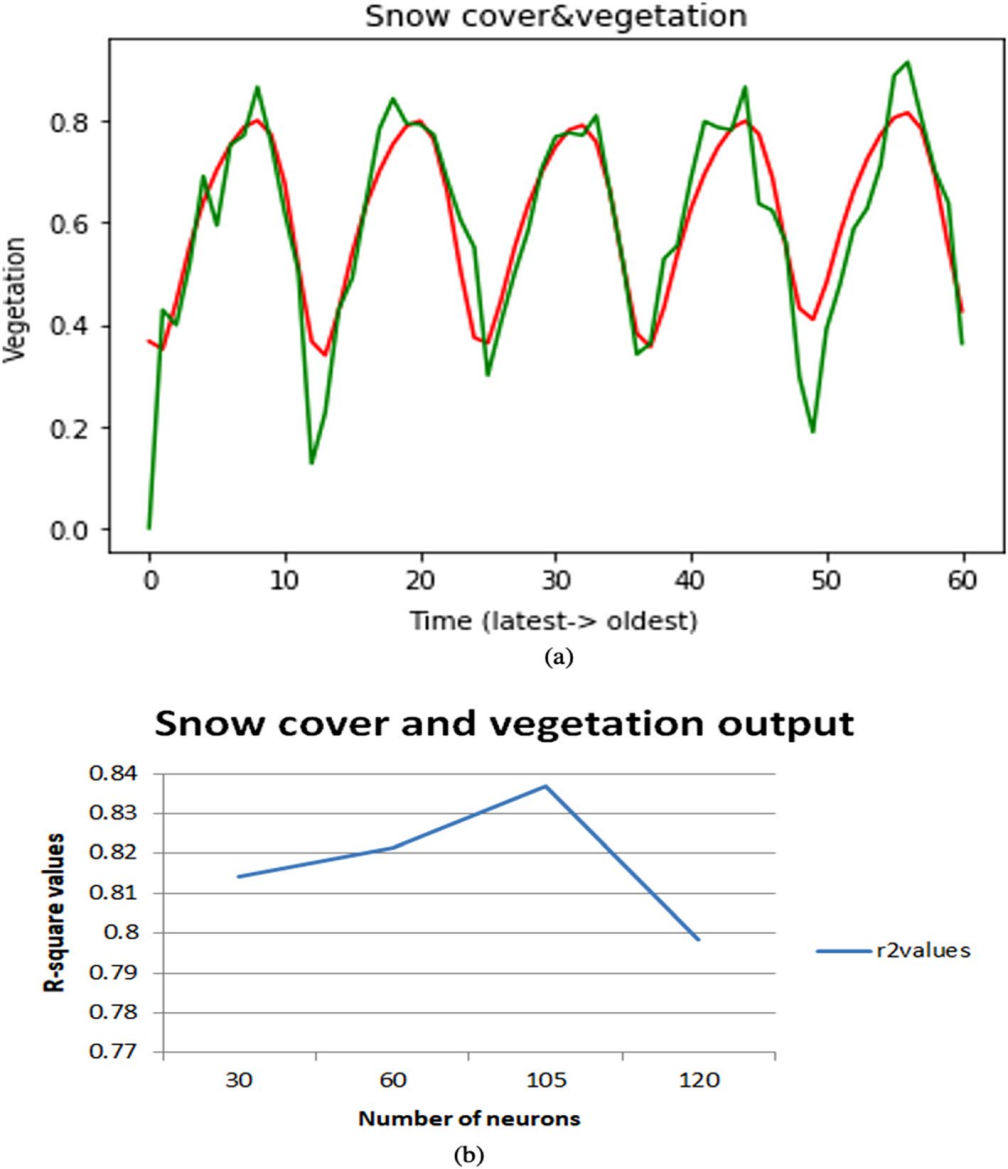
(**a**) Vegetation monthly as output on multiple combinations. (**b**) Changing the number of neurons and best r-square at 105 neurons.

In monthly forecasting, we will take the parameters based on monthly and take two parameters as input and one parameter as output. Here we are taking different combinations of parameters such as Temperature and Snow cover, Temperature and NDVI, Snow cover, and NDVI. To make all input features on a scale of 0 to 1, the input data is normalized using MinMaxScaler. As this is a problem of time series, if we need to forecast snow cover correctly then we need to consider temperature and snow cover from previous months as they are interrelated to each other accordingly for forecasting NDVI we consider these two combinations temperature-NDVI, snow cover-NDVI from previous months. A test dataset is created using 30% of the latest data along with the data created by going back to 60 months in the past for forecasting. This problem is a multivariate regression problem as it involves multiple input climate features. The model is compiled using Adam optimizer and the mean squared error is used as a loss function for this regression problem. To get the accurate values, we need to tune a set of hyperparameters i.e., number of neurons, optimizer, epochs, etc. In this monthly case, we had tuned the neurons to find the point where the model performs well. The present investigation used dropout with different values such as 0.25, 0.5 and 0.75 to avoid the overfitting, and it was observed that a value of 0.5 were more suited for dropout layer for all models based on performance, stability and efficiency of the models. Changing the number of neurons may also occur underfitting and overfitting, so we need to choose a suitable number of neurons. The R-square values of these results are for temperature and snow cover Fig. 5a,b it is 0.85 previous 0.51, for temperature and NDVI Fig. 6a,b it is 0.79 previous 0.48 and for snow cover and NDVI Fig. 7a,b it is 0.83 previous 0.48. If the R-square value is above 0.75 then it is considered a good model.

#### Seasonal

**Figure 8:**
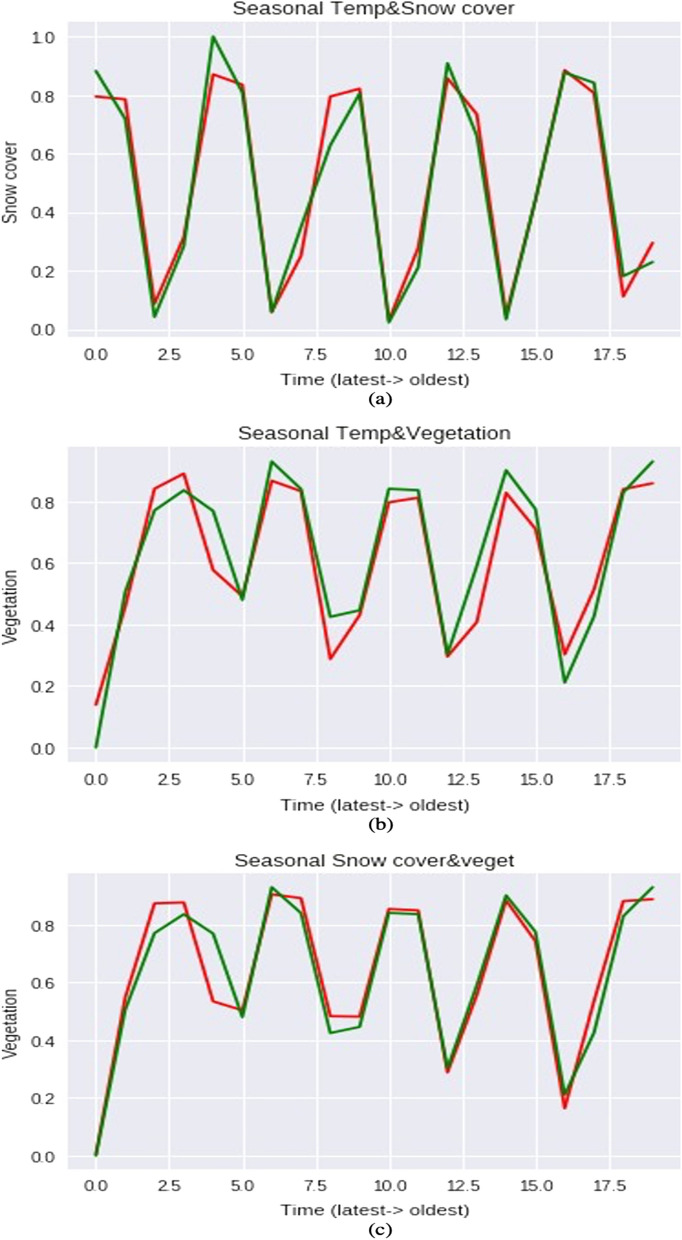
Seasonal output based on multiple combinations. (**a**) Seasonal temperature and snow cover. (**b**) Seasonal temperature and vegetation. (**c**) Seasonal snow cover and vegetation.

As we are dealing with temperature, snow cover, and vegetation which vary according to the season, we have divided a year into four seasons namely winter (December–February), pre-monsoon (March–May), monsoon (June–August), and post-monsoon (September–November). We converted monthly data into seasonal by taking the average of the months of that specific season for example to find the value of the temperature of monsoon season we took the average of 3 months in monsoon season. In this seasonal case, we have tuned the different optimizers i.e., ADAM, RMSProp, Adamax to get accurate results. In this method, the model best fits for ADAM optimizer as it combines the advantages of both AdaGrad and RMSProp and it achieves high R-square values for temperature and snow cover Fig. 8a it is 0.95, for temperature and NDVI Fig. 8b it is 0.89 and for snow cover and NDVI Fig. 8c, it is 0.92 (Table 1).

**Table 1:** R-square values for different optimizers.

Optimizer	Temperature and snow cover seasonal	Temperature and NDVI seasonal	Snow cover and NDVI seasonal
ADAM	0.95	0.89	0.92
RMSProp	0.93	0.88	0.90
Adamax	0.91	0.68	0.61

#### Annual

**Figure 9:**
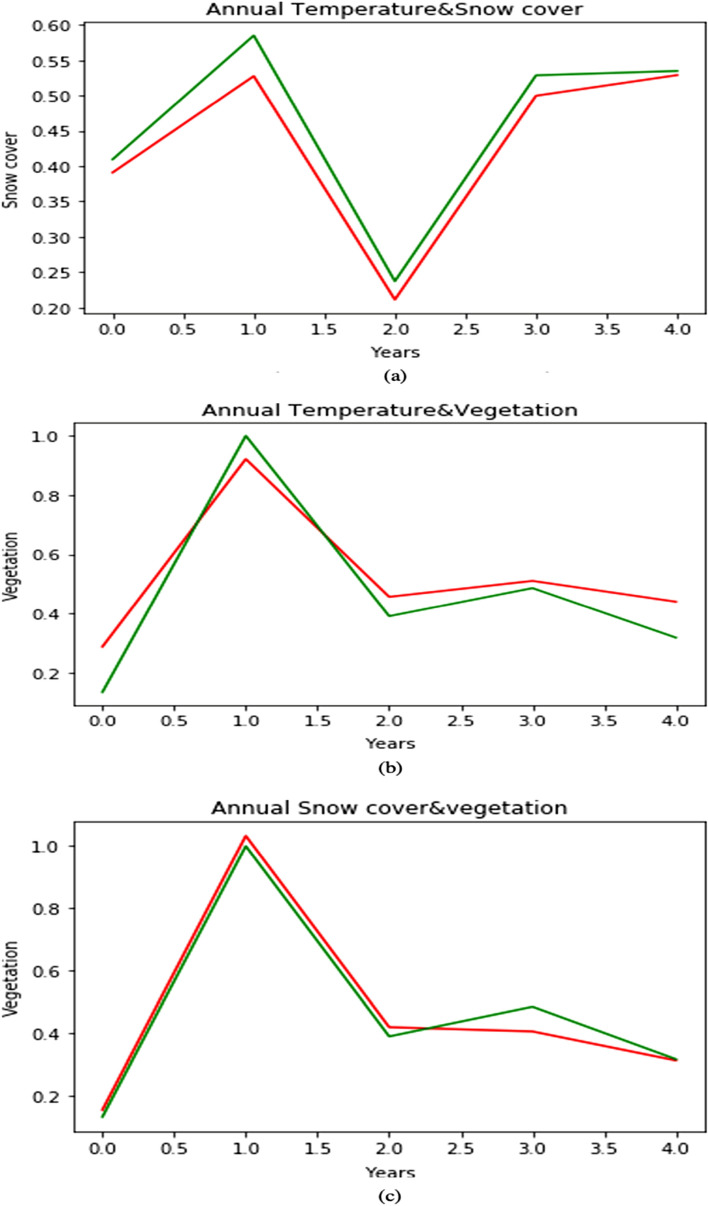
Ouput based on multiple combinations (**a**) Annual temperature and snow cover. (**b**) Annual temperature and vegetation. (**c**) Annual snow cover and vegetation.

The dataset consists of 17 years of data monthly-wise which we have averaged all months in that year to find annual data. The data is insufficient as it contains only 17 years of annual data. Compared to SVM, Decision tree and other classifiers, the neural networks may not categorise the data very well for less volume of the dataset. Therefore, the predicting model should be enough large to capture the interrelationships of the data along with features of the problem domain (e.g., number of categories). Initial layers of the model are used for capturing interrelationships among various parts of the input (i.e., patterns and edges). The features that can help to classify the desired outputs are captured by the output layer to yield the final decision. If the number of feature parameters and the amount of data required for forecasting is large, the chances for problems to have high complexity increases proportionally. As a result, it causes overfitting of the model. The R-square values obtained for temperature and snow cover Fig. 9a is 0.95, for temperature and vegetation Fig. 9b it is 0.97 and for snow cover and NDVI Fig. 9c, it is 0.98. So, we are not considering the annual case of our model because of overfitting.

#### LSTM forecasting

**Figure 10:**
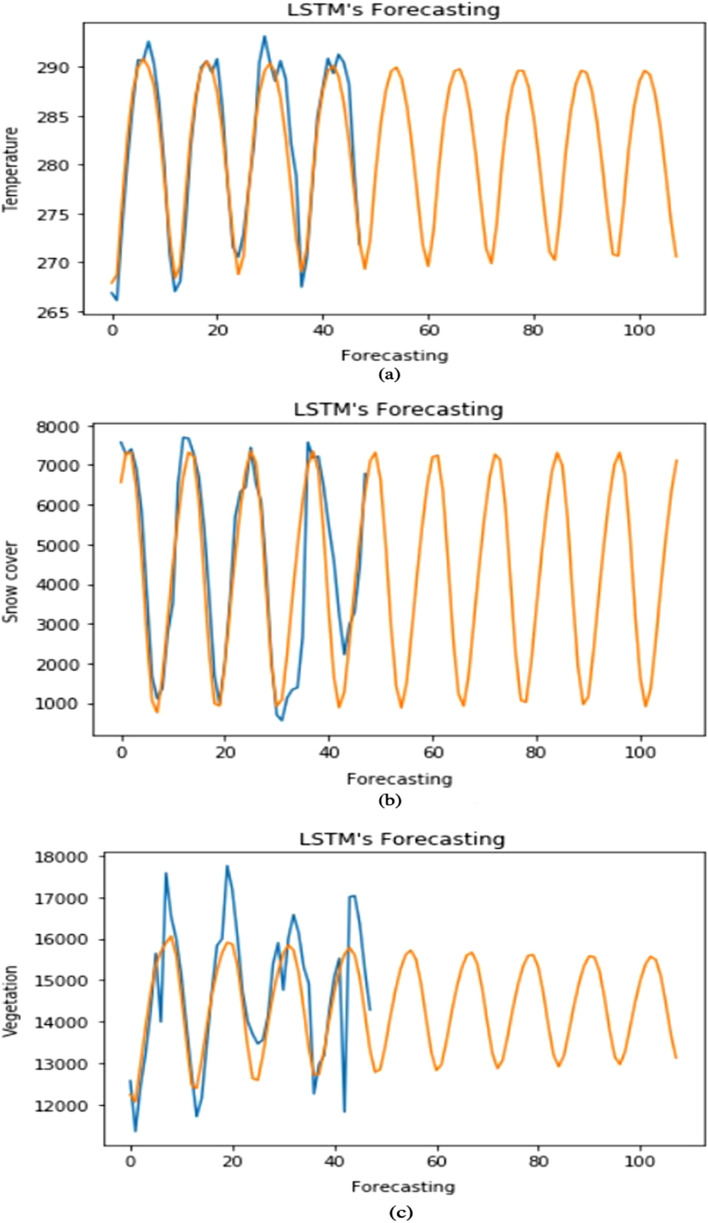
Forecasted values for next 7 years (**a**) Temperature. (**b**) Snow cover. (**c**) Vegetation.

Here we applied LSTM to forecast the data by taking one input and forecasting one output for the future seven years. The data is divided into 75% for training and 25% for testing the two-layered LSTM model along with a dense output layer, and then we forecasted the output step by step for test data and the model feeds the current forecasted value back into the input window by moving it one step forward to make forecasting at next time step and this is known as moving-forward window technique. The study uses a moving forward window of size 12. The 13th data point has been forecasted using the first 12 data inputs. And similarly, the 14th data point is forecasted using a window between 1 and 13 data inputs and the same procedure continue. For moving the window, panda’s shift function is used that shifts the entire column by the specified number. Similarly, the column is shifted up by 1 and then combined with the actual data. After fixing the size of the window, the first 12 columns of the table become input × 1, × 2,…, × 12 features and the 13th column becomes the target y. It is like if we are using LSTM for the NLP task then it looks like a fixed sequence of length 12 of a sentence containing 12 words each and trying to predict the 13th word. By using this method, the environmental factors individually for seven years are forecasted. Figure 10a–c represent forecasted values of temperature, snow cover, and NDVI for the future seven years.

## Trend test

Mann–Kendall test is used to analyze time-series data to find the weather trend is consistently increasing or decreasing. The data should have at least four samples however more the data points you have more likely we will get the true trend.

In this test, every value in the time series is compared with its preceding value which gives n(n-1)/2 data pairs where n is the total number of data points.

Sen’s estimator of the slope is the median of slopes of all data pairs.

$${\text{Q }} = \, \left( {{\text{Y}}_{j} - {\text{Y}}_{i} } \right)/\left( {{\text{j}} - {\text{i}}} \right)$$

Q is slope estimate, Y_*j*_ and Y_*i*_ are valued at times j and I where j is greater than i.

The confidence factor is a measure of the degree of likelihood that a rule is correct. It reflects the percentage of times that it has proven to be correct in the past. All the test values are calculated and result in a confidence factor of above 95% (Table 2).

**Table 2:** Trend test of forecasted values.

Time series	Temp. Q	Concentration trend	Snow cover Q	Concentration trend	NDVI Q	Concentration trend
Jan	0.35	** + **	34.03	** + **	57.15	** + **
Feb	0.12	** + **	− 77.16	**−**	123.50	** + **
Mar	− 0.21	**−**	− 203.29	**−**	115.03	** + **
Apr	− 0.34	**−**	− 282.68	−	92.88	** + **
May	− 0.32	**−**	− 250.43	**−**	71.99	** + **
June	− 0.24	**−**	− 125.80	**−**	46.83	** + **
July	− 0.15	**−**	45.21	** + **	10.18	** + **
Aug	− 0.06	**−**	183.01	** + **	− 43.47	**−**
Sept	0.04	** + **	225.75	** + **	− 108.32	**−**
Oct	0.15	** + **	207.57	** + **	− 153.26	**−**
Nov	0.26	** + **	166.82	** + **	− 139.74	**−**
Dec	0.34	** + **	106.80	** + **	− 52.87	**−**
Annual	0.00	**−**	2.48	** + **	1.66	** + **
Winter	0.27	** + **	21.22	** + **	42.59	** + **
Pre-monsoon	− 0.29	**−**	− 245.47	**−**	93.30	** + **
Monsoon	− 0.15	**−**	34.14	** + **	4.51	** + **
Post monsoon	0.15	** + **	200.05	** + **	− 133.77	**−**

## Conclusion

In this research work, a deep neural network LSTM model to make the forecasting of environmental factors have been used. The obtained results reflect that LSTM shows better performance compared to ANN in time series validation for individual parameters as deep learning provides a lot of benefits for time series forecasting. But even LSTM values are not efficient to consider the model for NDVI and snow cover, so as these parameters are interdependent to each other thus tried two input and one output model by tuning the various hyperparameters to get the better accuracy. Initially, we tried the different combinations of inputs like temperature-snow cover and NDVI-snow cover to improve the accuracy of snow cover and NDVI parameters. Later, tuned a different number of neurons and optimizers to find the optimal hyperparameters that give more efficient results. As the three parameters are seasonally dependent and applied the model on seasonal data it resulted in better R-square value compared to monthly. The R-square values obtained with annual data are high because of overfitting (due to fewer data). Thus, we cannot conclude that this is a good model for annual data. Then finally performed forecasting of future values using LSTM by moving-forward window technique for the next seven years i.e., 2018–2024. From trend test results forecasted values are of 95% confidence factor. So, the success of the model implies that the model could be used on other environment-related and time series problems and indicates that our LSTM model is robust. Google Colab and Keras provide an excellent environment to train and test models and can also be used to implement and run our proposed forecasting model. The future scope of the present investigation is to utilize the advanced deep learning models including^29–33^ for other regions of the world^34–36^. Another important future scope of the present investigation is to develop, host, and deploy near real time environmental monitoring status based on high end APIs^37,38^.

## Data Availability

The datasets used and/or analysed during the current study available from the corresponding author on reasonable request. Mohd Anul Haq (m.anul@mu.edu.sa) should be contacted if someone wants to request the data from this study.
